# Off Label Use of Botulinum Toxin in Children under Two Years of Age: A Systematic Review

**DOI:** 10.3390/toxins5010060

**Published:** 2013-01-07

**Authors:** Claudia Druschel, Henriette C. Althuizes, Julia F. Funk, Richard Placzek

**Affiliations:** Center for Musculoskeletal Surgery, Campus Virchow, Charité—University Medicine Berlin, Augustenburger Platz 1, Berlin D-13353, Germany; E-Mails: henriette-charlotte.althuizes@charite.de (H.C.A.); julia.funk@charite.de (J.F.F.); richard.placzek@charite.de (R.P.)

**Keywords:** botulinum toxin A, cerebral palsy, CP, multi-level treatment, key-muscle concept, infants, toddlers, motor development, motor milestones

## Abstract

The treatment of children with cerebral palsy with Botulinum Toxin is considered safe and effective, but is only approved for children older than two years of age. The effect of BoNT-A injection on juvenile skeletal muscle especially on neuromuscular junction density, distribution and morphology is poorly delineated and concerns of irreversible damage to the motor endplates especially in young children exist. In contrast, earlier treatment could be appropriate to improve the attainment of motor milestones and general motor development. This review systematically analyzes the evidence regarding this hypothesis. A database search, including PubMed and Medline databases, was performed and all randomized controlled trials (RCTs) comparing the efficacy of Botulinum Toxin in children younger than two years were identified. Two authors independently extracted the data and the methods of all identified trials were assessed. Three RCTs met the inclusion criteria. The results of the analysis revealed an improvement in spasticity of the upper and lower extremities as well as in the range of motion in the joints of the lower limbs. However, evidence of an improvement of general motor development could not be found, as the assessment of this area was not completely specified for this patient group. Based on available evidence it can not be concluded that Botulinum Toxin treatment in children younger than two years improves the achievement of motor milestones. However, there is evidence for the reduction of spasticity, avoiding contractures and delaying surgery. Due to some limitations, the results of this review should be cautiously interpreted. More studies, long-term follow up independent high-quality RCTs with effectiveness analyses are needed.

## 1. Introduction

In 1993, Koman reported the successful use of Botulinum Toxin (BoNT-A) in children with cerebral palsy (CP) for the first time [1]. Although BoNT-A is one of the most poisonous substances known, there has since been a growing interest in its therapeutic effects, above all in the muscle spasticity in children [2]. The preliminary studies were followed by a series of randomized trials [3] documenting mainly short-term reduction of muscle tone, prevention of muscle contractures and lever arm disease, as well as an improvement in function [4]. In addition to these positive effects, local intramuscular injection of the drug was demonstrated to be safe and rarely associated with side effects [5]. Overall, this data has led to a widespread introduction of BoNT-A in clinical practice, especially for treatment of children with CP [4]. Today, BoNT-A is licensed only for the treatment of spasticity in children two years of age or older, who have foot deformities due to persistent spasticity [6]. However, in the case of spasticity, very young children in the early stages of motor development are especially in need of intervention [7,8,9]. Besides abnormal muscle tone, direct consequences of the non-progressive damage to the central nervous system occurring in cerebral palsy include reduced or absent selective muscle control and disturbances in physiological balance mechanisms with limitations in motor development [8], particularly in upright stance and locomotion [10]. Overall, physiological motor development, characterized by the achievement of motor milestones, may be severely hampered and restricted [11,12]. Thus, securing the next milestone must be the focus of any therapy of young children with CP. Concerns about the high potential of possible side effects in such young children were alleviated by Pascual-Pascual *et al*., who demonstrated a good safety profile in infants younger than two years of age [13]. However, evidence concerning the benefits of early application with regard to functional motor development as well as delaying or avoiding surgery is rare. This paper presents the results of a systematic review of randomized controlled trials that assess the effect of BoNT-A treatment on motor development in children with cerebral palsy who were under two years old.

## 2. Results and Discussion

### 2.1. Results

**Figure 1:**
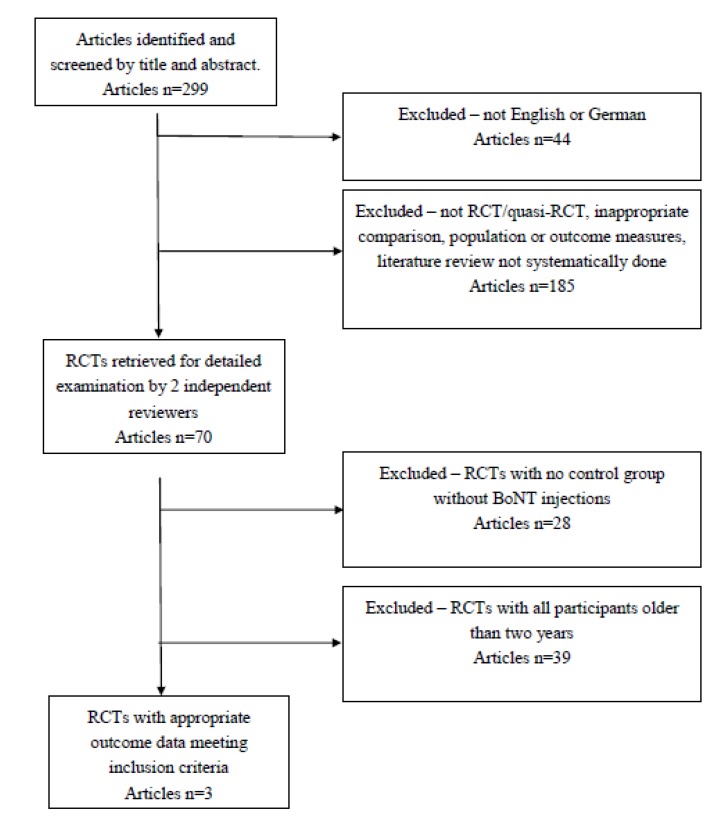
The flow chart demonstrates the number of articles and their exclusion after the defined criteria.

Of the 299 papers retrieved during the search, three studies were identified that met the criteria for appraisal (Figure 1). All of these were RCTs: Two compared BoNT-A injections combined with usual care or bracing versus usual care alone, and one compared BoNT-A injections combined with occupational therapy (OT) versus occupational therapy only. 

#### 2.1.1. Study Characteristics

The number of participants ranged from six to 47 children, both female and male, aged 11 months to 70 months with spastic CP (hemiplegia and diplegia). Regarding the age of the children, only Tedroff *et al.* included exclusively children under two years of age [4]. For the studies from Olesch *et al.* [14] and Graham *et al.* [15] no further itemized information regarding the number of treated children under two years was available in the published data. 

Olesch *et al.* [14] studied the effect of BoNT-A in combination with an intensive program of occupational therapy in the hemiplegic upper limb. The control group also received a twice-weekly program of OT for six weeks, but without any BoNT-A injection. Outcomes were assessed after 6 and 16 weeks. For primary outcomes, the assessment included the Canadian Occupational Performance Measure Change Score (COPM) and Goal Attainment Scale (GAS). For secondary outcomes, the Modified Tardieu Scale (MTS), the QUEST, and the Peabody Developmental Motor Scales-Fine Motor (PDMS-FM) were documented. 

Tedroff *et al.* [4] investigated the long-term effects of repeated BoNT-A injections in addition to daily stretching compared with a stretching program alone in the lower extremities. Assessments were performed at baseline, after one year and after an average of 3.5 years. Measurements included the Modified Ashworth Scale, ROM of the hip, knee and ankle, as well as the Gross Motor Function Measure (GMFM-66) and the Pediatric Evaluation of Disability Inventory (PEDI), wherein a division in secondary and primary outcomes was not undertaken. Furthermore, a three dimension gait analysis along a 10-m walkway was performed after the treatment and analyzed using the Gillette Gait Index (GGI). Children of appropriate age and development served as controls.

Graham *et al.* [15] compared BoNT-A injection in the lower limb in combination with a SWASH brace to a control group without any change in physiotherapy, seating or the orthoses. The primary study aim was a reduction in the progression of hip displacement measured by the migration percentage and the progression for surgery, while the secondary study aims were to examine safety, utility and compliance of this treatment regimen as well as the progression of hip displacement that led to surgery. Parameters were assessed at six month intervals over a study period of three years.

#### 2.1.2. Methodological Quality

All studies were classified as middle methodological quality RCTs (Table 1) ranging from seven to eight points out of a maximum possible score of 11 points using the PEDro (Physiotherapy Evidence Database) scale [16]. None of the included studies reported participant or therapist blinding. Furthermore, low methodological quality scores were caused by a lack of prognostic similarity at the baseline. Thus, the control group frequently exhibited a worse GMFCS level compared to the treatment group. 

**Table 1:** Methodologic quality assessment of included RCTs: PEDro scale.

Study	Olesch *et al.*	Tedroff *et al.*	Graham *et al.*
1. Specification of eligibility criteria	1	1	1
2. Random allocation	1	1	1
3. Concealed allocation	1	1	1
4. Prognostic similarity at baseline	1	0	0
5. participant blinding	0	0	0
6. therapist blinding	0	0	0
7. assessor blinding	0	0	1
8. >85% follow up of at least one key outcome	1	1	1
9. intention to treat analysis	1	1	1
10. between-group statistical comparison for at least one key outcome	1	1	1
11. Point estimates and measures of variability for at least one key outcome	1	1	1
Total score	8	7	8
% of maximum score	72.7	63.6	72.7

#### 2.1.3. Comparison of Interventions

BoNT dosages and injection modalities of the studies are summarized in Table 2. 

**Table 2:** Summary of dosage and injection modalities.

Study	Dosage	Drug	Injection site	Muscle identification	Time to reinjection	Anesthesia
Olesch *et al.*	0.5 U/kg to 2 U/kg body weight	Ona-BoNT-A (Botox^®^, Allergan)	M. biceps brachii (100%), M. pronator teres (91%), M. flex. carpi uln. (55%), M. flex. carpi rad. (18%), M. flex. dig. prof. (73%), M. flex. dig. sup. (73%) M. flex. poll. long. (45%) M. adductor pollicis (82%)	muscle stimulation	3 injections in 16 week cycles	general anesthetic
Tedroff *et al.*	6 U/kg body weight	Ona-BoNT-A (Botox^®^, Allergan)	M. gastrocnemius	palpation	2 injections with 6 month interval	without sedation
Graham *et al.*	6 U/kg body weight to a max. dose of 16 U/kg body weight	Ona-BoNT-A (Botox^®^, Allergan)	M. adductor longus M. gastrocnemius	palpation	repeated injections on the basis of clinical symptoms of spasticity	mask anesthesia

#### 2.1.4. Summary of Results

Table 3 displays each of the outcomes that were investigated in the studies, the component of health that would be affected, the measure that was used to evaluate the outcome, the result of that measure, and the inferential statistical data.

**Table 3:** The investigated outcomes of the studies, taking into account the component of health that would be affected, the measure that was used to evaluate the outcome, the result of that measure and the inferential statistical data.

Study	Outcome	Timing	*N*	Treatment	*N*	Control	*p*
				Result, Mean (±SD)		Result, Mean (±SD)	
Olesch *et al.*	MTS Elbow flexors	12 months	11	34.5 (48.0)	11	77.3 (56.2)	0.070
	Forearm pronators			22.7 (33.2)		72.7 (28.7)	0.001
	Wrist flexors			3.2 (7.2)		24.1 (28.5)	0.029
	QUEST Dissociated movements	12 months	11	79.9 (10.9)	11	74.9 (11.8)	n.r.
	Grasp			73.4 (11.0)		69.7 (14.1)	n.r.
	Weight bearing			88.9 (11.0)		86.1 (11.9)	n.r.
	Protective extension			75.8 (16.5)		60.9 (16.3)	n.r.
	Total score			79.6 (8.0)		72.9 (11.5)	0.129
	PDMS-FM	12 months	11	542.6 (36.2)	11	537.2 (37.2)	0.753
	COPM Performance	12 months	11	2.5 (1.0)	11	1.7 (0.6)	0.047
	Satisfaction			2.5 (1.1)		1.7 (0.9)	0.090
	GAS	12 months	11	55.8 (6.6)	11	48.8 (8.6)	0.047
				Change, Mean (±SD)		Change, Mean (±SD)	
Tedroff *et al.*	ROM Ankle joint	3.5 years	6	1.7	9	9	>0.05
	Knee joint			4		11	0.016
	Ashworth score Plantar flexor muscle tone	3.5 years	6	1	9	0.3	>0.05
	Knee flexor muscle tone			0.5		0.2	0.05
	GMFM-66	3.5 years	6	23.6	9	20.9	>0.05
	PEDI	3.5 years	6	n.r.	9	n.r	>0.05
Graham *et al.*	Migration percentage	3 years	43	2.6	42	5.5	0.05
	Progression to surgery	3 years	43	25.6	42	52.4	n.r

#### 2.1.5. Spasticity

Two studies measured signs of spasticity. Olesch *et al. *[14] demonstrated a significant reduction of spasticity in the treatment group for the forearm pronators and the wrist flexors. In the control group, no significant improvement was obvious. Focusing the lower limbs, Tedroff *et al.* [4] demonstrated a clear superiority in the treatment group with therapy of the plantar flexors compared to only minor changes in the control group in follow up compared to baseline. In the assessment of the knee flexor muscle tone no significant change between baseline and follow up occurred in either group. However, a statistically significant difference between the groups after 3.5 years was evident.

#### 2.1.6. Range of Movement

Only Tedroff *et al.* [4] demonstrated an initial non-significant increase of ankle dorsal extension, which returned to baseline levels during follow up sessions. Overall no significant difference between the treatment and control group appeared. However, a significant improvement in mobility was demonstrated under BoNT-A therapy compared to the control group in the range of motion in the knee joint.

#### 2.1.7. Motor Development

In terms of development of general motor skills, Olesch *et al.* [14] revealed no difference between the treatment and control group in the QUEST assessment. However, when considering the single domains of this tool, a progressive improvement in the grasp section was evident. Furthermore, the PDMS-FM demonstrated no significant difference between the different time points nor between the two groups. Tedroff *et al.* [4] also demonstrated no significant difference between therapy and control group by using the GMGM-66, but it significantly increased until the final follow up in both groups. A similar trend was found on the PEDI [4]. In the gait analyses, no significant difference between the two groups became evident.

#### 2.1.8. Other Aspects of Impairment

Olesch *et al.* [14] presented after 12 months a significant difference in COPM as well as GAS between therapy and control group [14]. Graham *et al.* evaluated the migration percentage for hip displacement [15]. This analysis revealed a significant difference between the two groups. However, when the analysis incorporated a weighting of the individual hips there was no significant difference. In the assessment of progression to surgery, children in the intervention group progressed at a lower rate than those of the control group [15].

#### 2.1.9. Adverse Effects and Complications

Table 4 summarized the adverse effects of the studies.

**Table 4:** Summary of the documented adverse effects in the studies.

Study	Symptoms	Percentage (%)	Course
Olesch *et al.*	maculopapular rash weakness of the index finger weakness in the finger flexors	27.3	completely resolved
Tedroff *et al.*	Weakness dysaesthesia of the skin pain at injection site	50.0	completely resolved
Graham *et al.*	major adverse effects (2 deaths)	6.0	
	minor adverse effects	16.0	completely resolved

### 2.2. Discussion

Although the FDA (U.S. Food and Drug Administration) approved BoNT-A in the management of strabismus, blepharospasm, hemifascial spasms and cervical dystonia, its use for spasticity in children with CP is restricted to foot deformities [9]. However, various randomized controlled trials and systematic reviews have demonstrated a decreased muscle tone and improved range of motion of joints in other indications, leading to recommendation of BoNT treatment in children with spasticity [17]. In order to evaluate the efficacy of BoNT-A treatment of children with cerebral palsy we performed this systematic review. 

Only three RCTs met the a priori inclusion criteria. The main limiting factor was the age of patients, to the extent that only one study, conducted in children less than two years of age [4], was identified. In order to reach a larger number of studies, RCTs not limited only to children younger than two years were included as well. In these studies, the proportion of children under two years of age could not be determined; therefore, an accurate correlation of BoNT’s therapeutic effect with respect to age is not available. Overall, 64 children with cerebral palsy were treated with BoNT-A injections, and 64 children were randomized to the control group receiving no BoNT injections. Due to a lack of participation and therapist blinding, which is generally difficult for ethical reasons, there is a limitation to the comparability of the baseline characteristics of both groups. Concerning the methodological quality of the included RCTs, all studies presented a middle quality according to the PEDro scale. Overall, our findings are mainly limited by the quality and number of the included studies, so that a potential publication bias cannot be excluded. 

In terms of the intention to treat, one study evaluated BoNT’s effect on the upper extremities, and two studies evaluated the lower extremities. These studies demonstrated superior results in the treatment group in reduction of spasticity in both the upper and lower extremities. This change was evident in both short- (12 months) and long-term (3.5 years) follow up [4,14]. These findings are consistent with studies performed in older children [18]. To assess the change in spasticity, the available studies used the MTS and MAS scale. Fosang *et al.* demonstrated that these methods can be used reliably, provided that scientists are allowed sufficient time for training and practice [19]. Thus, the obtained results of the studies must be evaluated as representative. In regard to the change in movement conditions, the analyses revealed indeterminate results, as only one study assessed this issue [4]. Despite no significant change being demonstrated in ankle dorsal extension, evidence was found for the avoidance of contractures in the treatment group by an increase of the popliteal angle [4]. 

In contrast to the positive effects on spasticity and mobility of the individual muscle groups, no improvement in overall motor development was demonstrated in the treatment group [4,14]. The authors utilized the QUEST and the PDMS-FM scales to evaluate the effects on the upper extremities [14]. The QUEST is a valid and reliable outcome measure, but validation studies using QUEST were performed with children aged 18 months to 8 years [20]; thus, the possibility of assessing effects in children younger than two years is limited. While no significant change was evident in the overall evaluation of the score, the analysis of the single domains demonstrated a significant improvement in the grasp section [14]. This distribution is based on the higher reliability of the domain scores [21]. In contrast, the PDMS-FM is standardized and normalized for the ages from birth through 72 months. The FM scale of the Peabody Developmental Motor Scales (PDMS) is one of the most commonly used standardized test for the assessment of fine motor (FM) development [22] due to its precise scoring system, large normative sample and high validity and reliability [23]. Nevertheless, a prospective study by Palisano *et al.* examined the validity of the PDMS for infants receiving physical therapy and found no proof of any changes over a six month period [24]. The authors therefore conclude that the PDMS is not recommended for evaluating the direct effect of physical therapy, but is recommended for providing a global measure of change in motor development [24]. This may explain the missing changes in the PDMS scale assessment of the BoNT treatment group. An analysis of the effect of the BoNT treatment on motor development of the lower extremity indeed demonstrated a significant improvement in the groups, but no statistically significant difference between the treatment and control group was observed [4,14]. Here, however, limitations of the selected assessment methods can be excluded due to a highreliability and validity in assessing the gross motor functions by the GMFM-66 [23]. In contrast, the application of the PEDI is only validated for ages 2 to 18 years for parent proxy-reporting [23]. Although the test has a high reliability and validity [25], its significance in terms of the herein considered issue is limited. Overall, failure to detect improvement with this general quantitative measurement could be caused by lack of sensitivity to a change at the individual level [26]. This conflict can be resolved by the assessment of functional treatment goals [27]. The evaluation of the family-based decision-making (COPM) as well as the individual goal-setting (GAS) clearly resulted in benefits for the BoNT injection group compared to the control group [14,15]. 

Considering further aspects of BoNT treatment, Graham *et al*. demonstrated a superiority of BoNT treatment by the evaluation of the migration percentage [15]. Although this was measured with use of a reliable protocol [15], the result of only 5.7% improvement in the treatment group must be considered critical in terms of clinical relevance. BoNT probably cannot prevent lateral hip displacement with later painful hip dislocation and arthritis [28] in children with CP, but might lead to a delay in the timing of surgery.

Analyzing the side effects of treatment in such young children, all available studies report adverse effects and complications with different degrees of severity [4,14,15]. In particular, the study of Tedroff *et al.* [4], which included children younger than two years of age, documented only minor complications. Furthermore, all studies demonstrated a mild and self-limiting character of these adverse effects [4,14,15]. Only Graham *et al.* [15] lists major adverse effects. Here, the combination of severe cerebral palsy, mask anesthesia and high dose BoNT therapy are shown to increase the risk of an acute respiratory event [29]. In contrast, Pascual-Pascual *et al.* emphasized a similar safety profile for infants younger than two years old and for older children in a retrospective analysis [15]. The effect of BoNT-A injection on juvenile skeletal muscle especially on neuromuscular junction density (NMJ), distribution and morphology is poorly delineated [30]. Experimental studies, primarily in rats, demonstrated an anatomical and physiological difference in neuromuscular junctions between juvenile and adult muscle, which may partially explain the variability in clinical results following BoNT-A injections [30]. Therefore, there are concerns that BoNT-A injections in such an early stage could possibly interfere the maturation process of the motor endplates [30,31]. Knowing that children with CP are weak [32], damaging the motor endplates in an irreversible way should be avoided by all costs.

## 3. Experimental Section

### 3.1. Search Strategy

We performed a computer-aided search of the following databases in July 2012: PubMed and Medline. The literature search was limited to published studies where full-text was available in English or German. The search used Medical Subject Headings (MeSH) terms and specific words within the text: “children”, “toddler”, “cerebral palsy”, “spasticity”, “Botulinum Toxin”, “Botox” and “randomized controlled trial”. The databases were searched within a time-frame from July 1993 until July 2012 because randomized controlled trials (RCTs) for the treatment of spasticity using Botulinum Toxin of children with CP were first reported in July 1993 [33]. We also undertook additional screenings of reference lists.

### 3.2. Information Sources

Prof. Dr. Graham was asked to specify about the age of the included children in the paper.

### 3.3. Eligibility Criteria and Study Selection

This review only included studies in which participants were children under the age of two years with CP who had received BoNT-A injections to either their upper or lower extremities. Because there was only one study that solely included children aged under two years, studies additionally including children over two years of age were included. Participants had to display spasticity in at least one extremity. The intervention had to include injections of Ona-BoNT-A and Abo-NoNT-A (Botox^®^ or Dysport^®^) into at least one affected muscle of the upper or lower limbs. Additional non-pharmacological treatments were acceptable. To be included, studies had to be randomized controlled trials including a control group without any BoNT treatment. Non-randomized trials and quasi-randomized trials were excluded. Furthermore, all randomized controlled trials had to measure one primary outcome including tasking function, gait pattern or gait parameters. Two reviewers independently reviewed titles and abstracts of articles retrieved using the aforementioned search strategy. Trials that failed to meet the inclusion criteria were not reviewed in their full extent by the two reviewers. Three trials were eligible for inclusion.

### 3.4. Data Extraction

Details of the methodologies and populations were summarized for all trials (Table 5). Methodological quality of the included trials was evaluated by using the PEDro (Physiotherapy Evidence Database) scale [16]. The following trial details were extracted from the publications: the description of the study population (number, age, sex of the participants, reason and type of spasticity), the description of the intervention (frequency and dosage of BoNT-A, injected muscle, additional therapy), the comparison treatment, the functional-related outcome measures used and the main results of the study. 

**Table 5:** Study characteristics and methods of RCTs.

Study	Design	Diagnosis	Age	Treatment	*n*	Control	*n*
Olesch *et al.*	SB RCT	CP	1 year 10 months to 4 year 10 months	BoNT-A and OT	11	OT	11
Tedroff *et al.*	SB RCT	CP	11 months to 1 year 11 month	BoNT-A and ST	6	Control	9
Graham *et al.*	RCT	CP	1 year to 5 year	BoNT-A and Bracing	47	Control	44

ST = stretching; OT = occupational therapy; SB = single blinded.

### 3.5. Analysis

The acquired data of our study showed no common denominator in essential data required for a proper assessment in a meta-analysis.

## 4. Conclusion and Direction of Further Research

Overall, this review suggests an advantage of the treatment with Botulinum Toxin in reducing spasticity, avoiding contractures and delaying surgery in children younger than two years. However, clear evidence regarding the improvement in general motor development cannot be derived. This demonstrates the need for further randomized controlled trials analyzing this issue as well as the application of sophisticated measuring methods, which are reliable and valid for children of this age group.
